# Knockdown of Rab7a suppresses the proliferation, migration, and xenograft tumor growth of breast cancer cells

**DOI:** 10.1042/BSR20180480

**Published:** 2019-02-05

**Authors:** Jiming Xie, Yan Yan, Fang Liu, Hongbin Kang, Fengying Xu, Weili Xiao, Haiyan Wang, Yuzhen Wang

**Affiliations:** 1 Clinical Laboratory, Inner Mongolia People’s Hospital, Hohhot 010010, P.R. China; 2 College of Life Science, Inner Mongolia Agricultural University, Hohhot 010018, P.R. China; 3 Department of General Surgery, The Affiliated Hospital of Inner Mongolia Medical University, Hohhot 010010, P.R. China; 4 Inner Mongolia Medical University, Hohhot 010110, P.R. China

**Keywords:** apoptosis, cell cycle arrest, migration, proliferation, Rab7a

## Abstract

Breast cancer is a common invasive cancer in women. Ras-related protein Rab-7a (Rab7a) is involved in late endocytic trafficking, while its role in breast cancer is largely unclear. In the present study, we investigated the role of Rab7a in breast cancer. Comparing with adjacent breast tissues, Rab7a expression was increased in breast cancer tissues. Using lentivirus-mediated knockdown strategy, we found that Rab7a silencing inhibited the proliferation and colony formation of MDA-MB-231 cells. Apoptosis and G_2_ cell cycle arrest were induced in Rab7a knockdown. By contrast, Rab7a suppressed the apoptosis and promoted proliferation and colony formation of MCF-7 cells. The migration of MDA-MB-231 cells was suppressed by Rab7a knockdown. *In vivo*, depletion of Rab7a inhibited the xenograft tumor development of MDA-MB-231 cells. Altogether, our results highlight the novel function of Rab7a in the proliferation, invasion, and xenograft tumor development of breast cancer cells.

## Introduction

Breast cancer is the commonest malignancy and the leading cause of cancer-related deaths in women worldwide [1]. Estrogen is a major risk factor for breast cancer and a large amount of the patients are closely associated with estrogen receptor or progesterone receptor [2,3]. Based on this mechanism, tamoxifen which blocks the binding of estrogen with its receptor is considered as a promising hormonal therapy [4]. However, numerous health problems emerge as this treatment enlarges the risk of thromboembolism and endometrial cancer [5]. Other therapeutic options including surgery, radiation, and chemotherapy are widely used while they exhibit poor prognosis. Therefore, understanding the common molecular events may help us gain insight into the therapeutic strategies for this disease.

Ras-related protein Rab-7a (Rab7a) and Rab7b, two homologous isoforms belonging to Rab7a family, display high sequence similarity [6]. Unlike Rab7b which is in charge of trafficking from endosomes to Golgi apparatus, Rab7a is located in late endosome and controls transport to endocytic degradative compartments [7–11]. It is required for maturation of lysosomes, phagosomes, and autophagosomes, and has been shown to regulate cell survival and apoptosis [12]. Recently, some studies have reported the role of Rab7a in cancer. Vimentin, an indicator of epithelial–mesenchymal transition, is regulated by Rab7a in cancer cells [13,14]. Vorinostat and simvastatin synergistically inhibit the tumor development of triple-negative breast cancer cells by inhibiting Rab7 prenylation [15]. Although some studies have illustrated its involvement in breast cancer, direct evidence is very limited on its significance in breast cancer development.

Here we identified Rab7a as an oncogene in breast cancer. Breast cancer tissues showed enhanced Rab7a expression compared with adjacent breast tissues. Knockdown of Rab7a blunted the proliferation, colony formation, and xenografted tumorigenesis of MDA-MB-231 cells. Rab7a silencing also suppressed the migration capacity. In contrast, Rab7a overexpression accelerated the proliferation and growth of MCF-7 cells. Mechanistically, Rab7a knockdown increased the apoptosis and decreased G_2_ cell cycle progression of MDA-MB-231 cells. At the molecular level, 262 genes were up-regulated and 372 genes were down-regulated after Rab7a knockdown.

## Materials and methods

### Patient information

All 101 patients of breast cancer and 28 normal breast tissues were enrolled from 2013 to 2015 at Inner Mongolia People’s Hospital. The study was approved by the Ethics Committee of Inner Mongolia People’s Hospital. Written informed consent was obtained from all the patients.

### Cell lines and cell culture

Human normal breast HMepC cells and breast cancer cells ZR-75-30, MCF-7, T-47D, MDA-MB-231, and HCC-1937 were from American Type Culture Collection and cultured in 1640 medium (Invitrogen), supplemented with 10% FBS (Thermo) and 1% penicillin/streptomycin (Corning). All the cells were maintained at 37°C with 5% CO_2_.

### Immunohistochemistry

Formalin-fixed, paraffin-embedded (FFPE) sections (4 μm) of breast cancer or normal tissues were deparaffinized in xylene and hydrated in graded alcohol. These slides were subjected to Hematoxylin and Eosin staining (HE) and immunohistochemical analysis of Rab7a. For immunohistochemical assay, citrate buffer (pH = 6) was used for antigen retrieval. Endogenous peroxidase was blocked by 3% hydrogen peroxide for 15 min. Slides were blocked with 10% goat serum for 60 min. Then, the sections were incubated with Rab7a antibody at 4°C overnight and with secondary antibodies at room temperature for 30 min. AEC was used as a chromogen, and slides were sealed with balsam.

### Total mRNA isolation and quantitative real-time PCR (qRT-PCR)

Breast cancer cells were lysed in TRIzol reagent (Invitrogen). Total RNA was isolated from these cells using RNeasy Mini kit (Qiagen). One microgram of the total RNA was reverse-transcribed using ReverTra Ace® qPCR RT Master Mix with gDNA Remover (TOYOBO), according to the manufacturer’s instructions. Quantitative real-time PCR (qRT-PCR) experiments were performed using TransStart Top Green qPCR SuperMix (TransGen Biotech) on an IQ-5 machine. Glyceraldehyde-3-phosphate dehydrogenase (GAPDH) serves as an internal control. The real-time PCR primers were listed as followed: Rab7a forward: GTCGGGAAGACATCACTCA and reverse: CTAGCCTGTCATCCACCAT GAPDH forward: 5′- TGACTTCAACAGCGACACCCA-3′ and reverse: 5′- CACCCTGTTGCTGTAGCCAAA-3′.

### Western blot

Breast cancer cells were lysed in lysis buffer (2 g SDS, 1.55 g DTT, 6 ml Tris (1 M, pH 6.8), 10 ml glycerol, and ddH_2_O up to 100 ml). Fifty to eighty micrograms of the total protein was separated on an SDS/PAGE (12% gel), and then was transferred on to PVDF membrane (Millipore). The membranes were blocked with 5% skim milk dissolved in PBS and Tween 20 (PBST) for 1 h at room temperature and incubated with primary antibodies overnight at 4°C. After incubating with horseradish peroxidase-conjugated secondary antibodies at room temperature for 2 h, the immune activity on the membranes was detected with ECL-Plus kit (Amersham Biosciences). Antibody against eukaryotic translation initiation factor 4E (eIF4E) (#2067), AMPKα (#5832), and ribosomal protein S6 kinase B1 (RPS6KB1) (#2708) were purchased from Cell Signaling Technology. Antibody against GAPDH was purchased from Santa Cruz Biotechnology (SC-32233). All the secondary antibodies were from Santa Cruz Biotechnology.

### Lentivirus-mediated Rab7a knockdown in MDA-MB-231 cells

Rab7a was knocked down using a pGCSIL-GFP lentivirus vector. In brief, shRNA against Rab7a (KD1: 5′-ACGAATTTCCTGAACCTAT-3′, KD2: 5′-AACAAGATTGACCTCGAAA-3′, KD3: 5′-CACAATAGGAGCTGACTTT-3′, KD4: 5′-CATTTGTTGTGTTGGGAAA-3′) or NC (5′-TTCTCCGAACGTGTCACGT-3′) was cloned into pGCSIL-GFP vector by GeneChem Corporation (Shanghai, China). The KD-1, 2, 3, or 4 represented different target shRNA of Rab7a. qRT-PCR assay was used to determine the knockdown efficiency. In brief, mixture of pGCSIL-shRNA-GFP (pGCSIL-GFP, stably expressed shRNA fused with a GFP marker), pHelper1.0 (gag/pol element) and Helper2.0 (VSVG element) were co-transfected into 293T cells using Lipofectamine™ 2000 (Invitrogen) according to the manufacturer’s instructions. Two days after transfection, lentivirus supernatants were harvested and filtered through a 0.45-μm PVDF membrane. Then the lentivirus supernatants were used to infect indicated cells. Non-transfected (Ctrl) and control-transfected (NC) MDA-MB-231 cells served as control cells.

### Rab7a ectopic expression in MCF-7 cells

The coding sequence of Rab7a was inserted into pBABE-puro vector. pBABE-puro vector and packaging vector PIK were co-transfected into 293T cells using Lipofectamine 2000 (Invitrogen). Forty-eight hours later, viral supernatants were collected and filtered through a 0.45-μm filter and subjected to infection of MCF-7 cells. Puromycin was used to select stable cell lines.

### Cell proliferation assay

MTT assay was used to detect the proliferation rate**.** For clarity, a total of 4000 shCtrl or shRab7a MDA-MB-231 cells, and 3500 Ctrl or Rab7a overexpressed MCF-7 cells were seeded in 96-well plates and the cell viability of 24, 48, 72, 96, or 120 h was measured. MTT solution (5 mg/ml) was added into each well and incubated at 37°C for 3 h. Then the culture medium with MTT solution was removed and 150 μl DMSO was added. The optical density (OD) of 490 nm was detected on a microplate reader.

### Colony formation assay

Equal number of MDA-MB-231 cells expressing shCtrl or shRab7a lentivirus, and Ctrl or Rab7a overexpressed MCF-7 cells were seeded in six-well plates (400 cells/well) and cultured at 37°C. Colonies were formed after maintaining 14 days. For analysis of the colonies, the cells in six-well plates were fixed with methanol for 0.5 h and stained with 0.5% Crystal Violet solution for 15 min. The colony numbers were determined using fluorescence microscopy (Olympus).

### Cell cycle assay

Cell cycle progression was determined using propidium iodide (PI, Sigma–Aldrich) staining. MDA-MB-231 cells expressing shCtrl lentivirus or shRab7a lentivirus were seeded in six-well culture plates. Cell cycle was analyzed by PI staining. PI absorbance of indicated cells was determined by FACS on a flow cytometry.

### Apoptosis assay

V-APC apoptosis detection kit (Ebioscience) was used to determine the apoptosis of shCtrl or shRab7a MDA-MB-231 cells, and Rab7a overexpressing or Ctrl MCF-7 cells, following the manufacturer’s protocol. The cells were washed with PBS and resuspended at a density of 1 × 10^6^ per ml in staining buffer. Five microliters of annexin V-APC was added and the mixed solution was incubated for 15 min at room temperature. Then they were subjected to flow cytometry analysis (FACSCalibur, Becton-Dickinson).

### Cell invasion analysis

Matrigel-coated 8.0-μm filter invasion chambers (BD Biosciences, U.S.A.) were used to determine the cell invasion of shCtrl or shRab7a MDA-MB-231 cells. After incubating at 37°C with 5% CO_2_ for 24 h, cells were removed from the upper surface of the membrane by cotton tips. The migrant cells attached to the lower surface were stained with Crystal Violet for 30 min. The membranes were washed with PBS, and then the attached cells on the filter were detected using an optical microscope (magnification: 200×).

### Xenograft tumor formation assay

A total of 1 × 10^7^ shCtrl or shRab7a MDA-MB-231 cells were subcutaneously implanted into the right armpit of the 4-week-old male mice. Diameters of xenograft tumors were measured by a slide caliper rule. The tumor volume was determined by the following formula: v = 0.5ab^2^ (a, long diameter of the tumor; b, short diameter of the tumor; and v, volume). The study was approved by the Ethics Committee of Inner Mongolia People’s Hospital.

### Statistical analysis

GraphPad Prism 6.0 software was used to determine the statistical analysis. The data as shown were mean ± S.E.M. of at least three independent repeats. Difference between two groups was analyzed by Student’s *t* tests. One-way ANOVA was used when more than two groups as shown in Figures 2–6. *P*-value less than 0.05 was defined as statistically significant.

## Results

### Rab7a is highly expressed in breast cancer tissues

**Figure 1:**
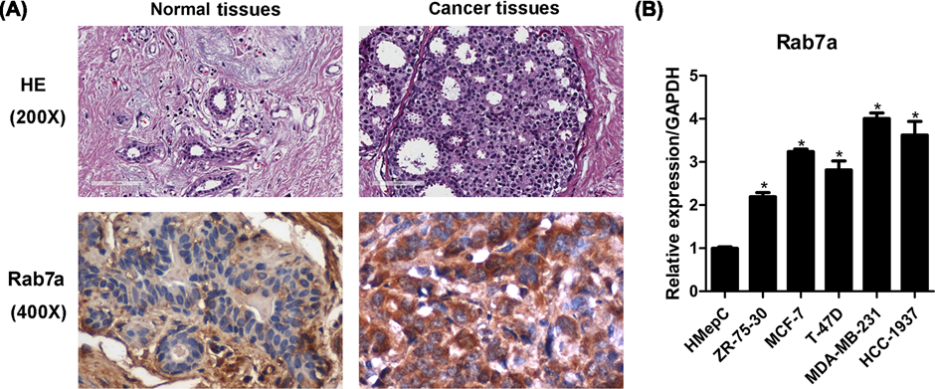
Rab7a is up-regulated in breast cancer tissues (**A**) HE staining and immunohistochemical staining of Rab7a in breast cancer (*n*=101) and normal breast tissues (*n*=28). (**B**) qRT-PCR results of *Rab7a* mRNA expression in normal breast cells HMepC and breast cancer cells ZR-75-30, MCF-7, T-47D, MDA-MB-23, and HCC-1937. *P<0.05.

To investigate the clinical relevance of Rab7a in breast cancer, we collected breast cancer samples and checked Rab7a expression by immunohistochemical staining. We found that Rab7a was up-regulated in breast cancer tissues compared with adjacent normal tissues (Figure 1A and Table 1). mRNA expression of Rab7a was detected in normal breast cell line HMepC and in various breast cancer cells, including ZR-75-30, MCF-7, T-47D, MDA-MB-231, and HCC-1937 cells. We found that *Rab7a* mRNA level was higher in these breast cancer cells comparing with HMepC cells (Figure 1B). Collectively, Rab7a is a potential biomarker for breast cancer.

**Table 1:** Breast cancer tissues show higher expression of Rab7A

Type of tissue	Number of cases	Rab7A expression				
		−	+	++	+++	*P*-value
Cancer tissues	101	0	18	32	51	0.0001
Normal tissues	28	7	9	6	6	

‘−’ negative, ‘+’ positive, ‘++’ moderately positive, ‘+++’ significantly positive.

### Rab7a knockdown suppresses the cell proliferation and growth of MDA-MB-231 cells

**Figure 2:**
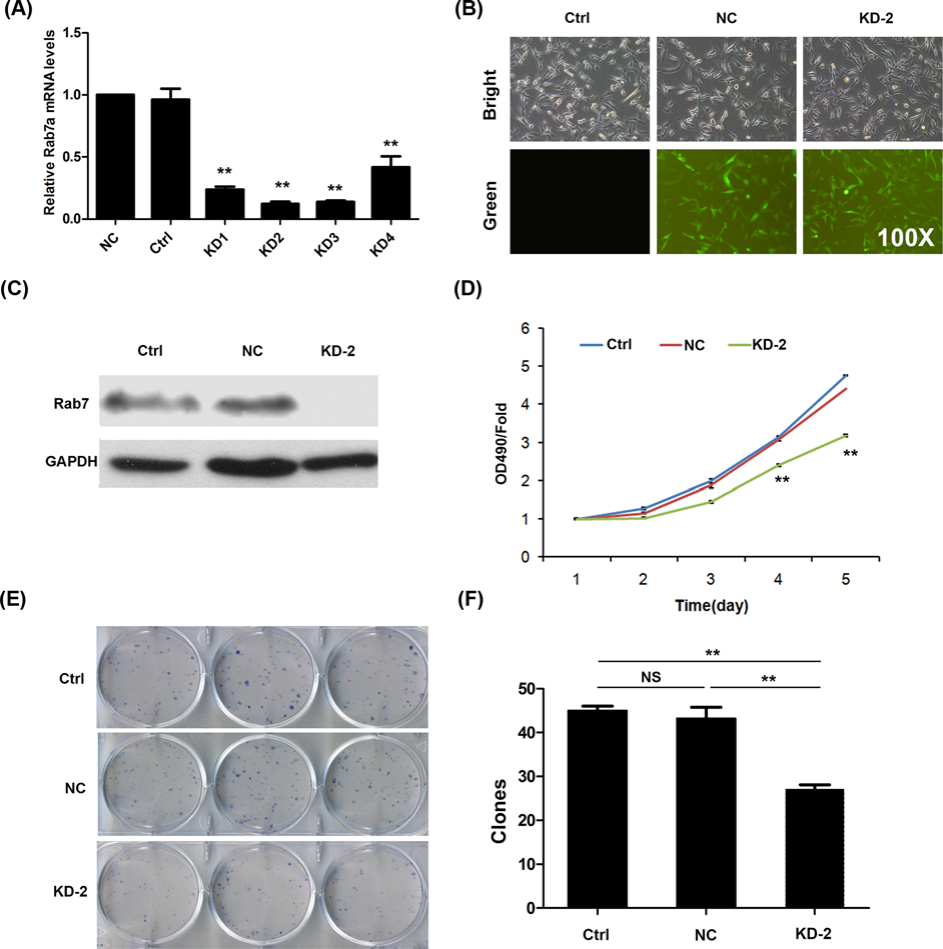
Efficient knockdown of Rab7a leads to suppressed cell viability and growth of MDA-MB-231 cells (**A**) mRNA expression of Rab7a in MDA-MB-231 cells transfected without shRNA lentivirus (Ctrl), shNC lentivirus (NC), or shRab7a lentivirus (KD1, 2, 3, and 4). **P<0.01; *n*=3. (**B**) Green fluorescence images of MDA-MB-231 cells transfected without shRNA lentivirus (Ctrl), shNC lentivirus (NC), or shRab7a lentivirus (KD2). (**C**) Western blots of Rab7a in cells as shown in (B). (**D**) Cell viability of Ctrl, shNC, and shRab7a (KD2) MDA-MB-231 cells was determined by MTT assay from day 1 to 5. **P<0.01; *n*=5. (**E**) Colony formation of indicated cells. (**F**) Quantitative results of colony formation in (E). **P<0.01; *n*=3. Abbreviation: NS, no significance.

To determine the role of Rab7a in breast cancer, we silenced Rab7a using lentivirus-mediated knockdown strategy in MDA-MB-231 breast cancer cells. Four Rab7a knockdown MDA-MB-231 cells were constructed. Compared with NC, *Rab7a* mRNA level was lowest in KD2 clone, followed by KD1, 3, and 4 (Figure 2A). KD2 knockdown MDA-MB-231 cells exhibited high content of green fluorescence, which is an indicator of silencing efficiency (Figure 2B). Consistently, Western blot results also showed efficient silencing of Rab7a in KD2 MDA-MB-231 cells (Figure 2C).

**Figure 3:**
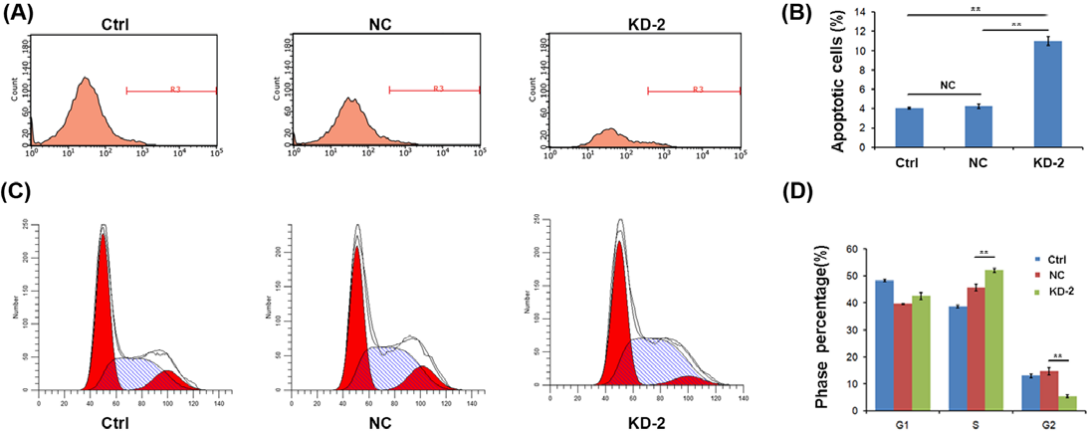
Rab7a silencing increases apoptosis and retards cell cycle progression of MDA-MB-231 cells (**A**,**B**) Flow cytometry analysis of cell cycle showed that Rab7a knockdown induced MDA-MB-231 cell cycle arrested at S-phase. **P<0.01; *n*=3. (**C**,**D**) Flow cytometry analysis of apoptosis revealed that Rab7a knockdown induced MDA-MB-231 cell apoptosis. **P<0.01; *n*=3. NC, negative control.

Next, we analyzed the effect of Rab7a silencing on breast cancer cell viability. Based on MTT assay, we found that Rab7a knockdown decreased the cell proliferation rate of MDA-MB-231 cells at days 4 and 5 (Figure 2D). There was no significant suppression from day 1 to 3 (Figure 2D). We also analyzed the cell growth by colony formation test. The results showed that Rab7a knockdown suppressed the colony formation capacity of MDA-MB-231 cells (Figure 3E,F). Taken together, Rab7a knockdown results in suppressed MDA-MB-231 cell proliferation and growth.

### Rab7a knockdown induces apoptosis and cell cycle arrest of MDA-MB-231 cells

Cancer cell proliferates faster partly depending on decreased apoptosis and accelerated cell cycle progression. We next analyzed the apoptosis in shCtrl or shRab7a MDA-MB-231 cells. ShRab7a MDA-MB-231 cells exhibited increased apoptosis (Figure 3A,B). Additionally, cell cycle division was also determined. Rab7a knockdown led to decreased G_2_ phase and increased S-phase distribution of the cell cycle, while the distribution of G_1_ phase remained at minimal change (Figure 3C,D), suggesting that cell cycle was arrested at the S-phase in shRab7a MDA-MB-231 cells. Taken together, Rab7a silencing in MDA-MB-231 cells results in enhanced apoptosis and cell cycle arrest.

### Rab7a overexpression suppresses the apoptosis and promotes the proliferation and growth of MCF-7 cells

**Figure 4:**
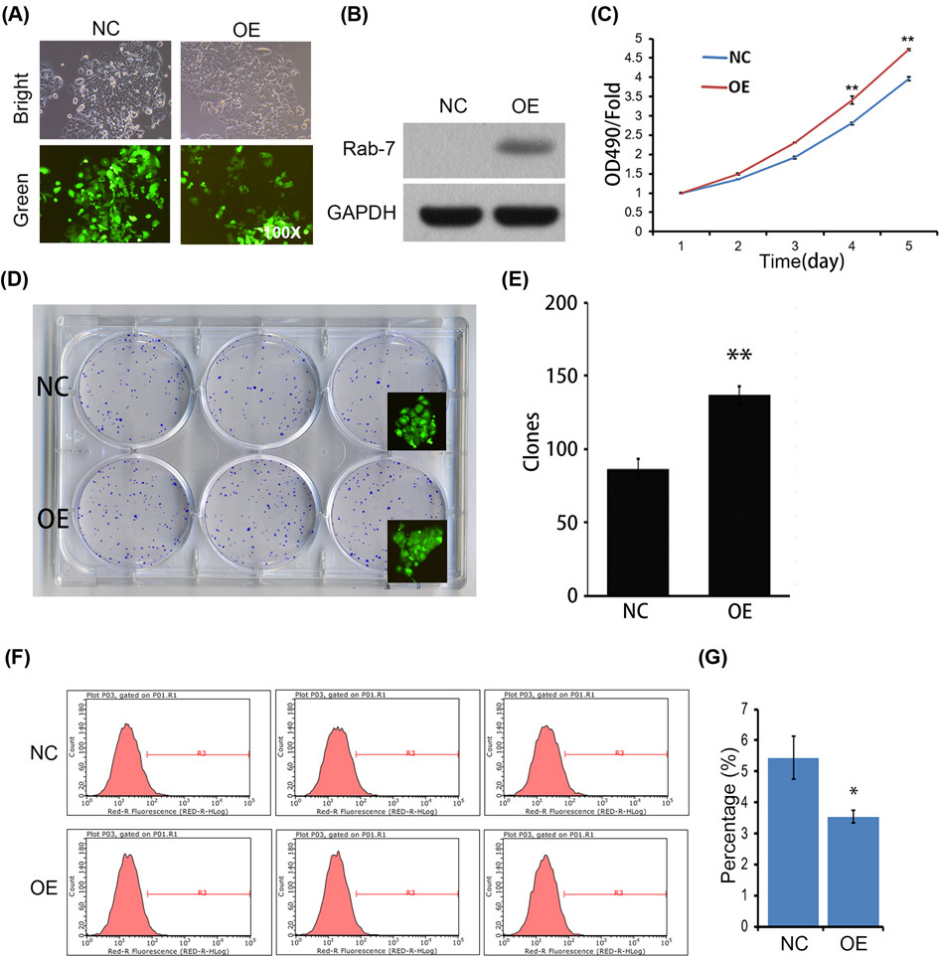
Rab7a overexpression reduces the apoptosis and promotes the proliferation and growth of MCF-7 cells (**A**) Green fluorescence images of Rab7a overexpressed (OE) and Ctrl (NC) MCF-7 cells. (**B**) Western blots of Rab7a in cells as shown in (A). (**C**) Cell viability of Rab7a OE and Ctrl (NC) MCF-7 cells was determined by MTT assay from day 1 to 5. **P<0.01; *n*=5. (**D**) Colony formation of indicated cells. (**E**) Quantitative results of colony formation in (D). **P<0.01; *n*=3. (**F**,**G**) Flow cytometry analysis of apoptosis revealed that Rab7a overexpression suppressed MCF-7 cell apoptosis. *P<0.05; *n*=3.

To confirm our findings, we next overexpressed Rab7a in MCF-7 cells. We found that Rab7a ectopic expression promoted the proliferation and colony formation of MCF-7 cells (Figure 4A–E). In addition, apoptosis was reduced in Rab7a overexpressed MCF-7 cells compared with Ctrl cells (Figure 4F,G). We suggest that Rab7a inhibits the apoptosis and promotes the proliferation and growth of breast cancer cells.

### Rab7a knockdown inhibits the invasion of MDA-MB-231 cells

**Figure 5:**
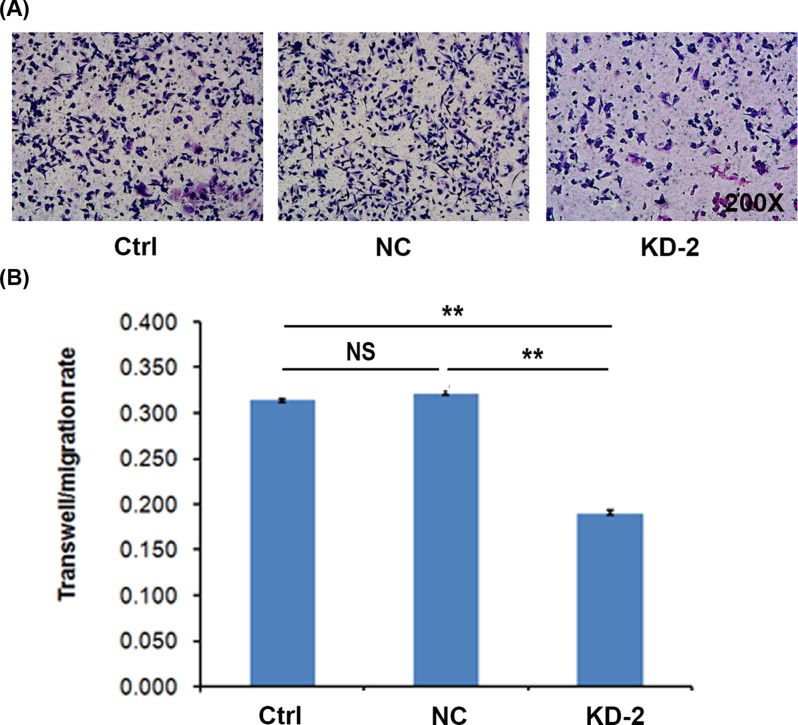
Rab7a knockdown suppresses cell invasion (**A**) Cell invasion of Ctrl, shNC, and shRab7a MDA-MB-231 cells was determined by Transwell assay. (**B**) Quantitation results of cell invasion. **P<0.01; *n*=3. Abbreviation: NS, no significance.

We also investigated the role of Rab7a in cell invasion of MDA-MB-231 cells by Transwell assays. Our results showed that Rab7a silencing suppressed the migration ability of MDA-MB-231 cells (Figure 5A). Quantitative results were consistent (Figure 5B). These findings indicated that Rab7a might be critical for the invasion of MDA-MB-231 cells.

### Rab7a silencing suppresses the xenograft tumor development in MDA-MB-231 cells

**Figure 6:**
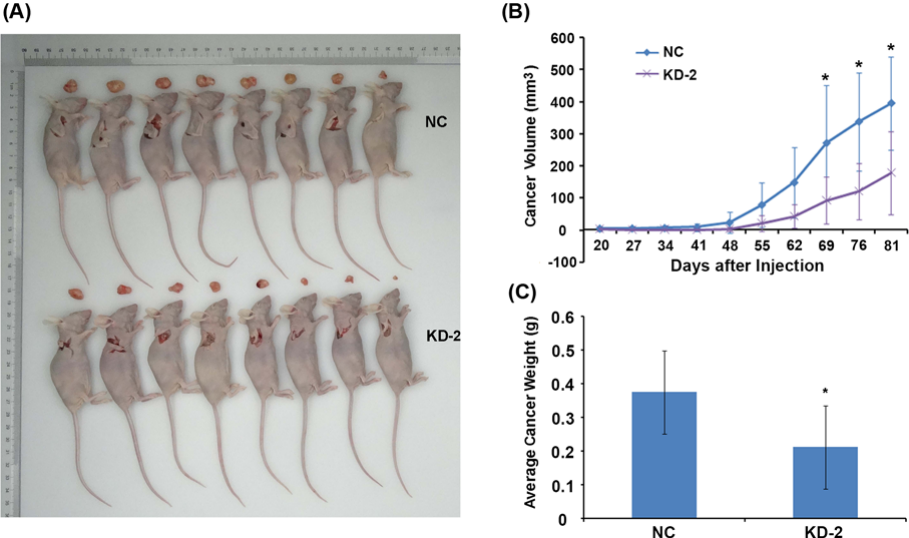
Rab7a knockdown suppresses xenograft tumor growth in MDA-MB-231 cells in nude mice (**A**) Photographs of xenograft tumors of shNC and shRab7a MDA-MB-231 cells by the time of day 81, *n*=8. (**B**) The volume of xenograft tumors derived from shNC and shRab7a MDA-MB-231 cells was checked from day 20 to 81. *P<0.05, *n*=8. (**C**) The weight of xenograft tumors of shNC and shRab7a MDA-MB-231 cells by the time of day 81. *P<0.05, *n*=8.

To explore the role of Rab7a knockdown in tumor development, we subcutaneously implanted the shCtrl or shRab7a MDA-MB-231 cells into the nude mice. Tumor volume was monitored and the results showed that Rab7a silencing moderately suppressed the tumor development of MDA-MB-231 cells in nude mice (Figure 6A,B). By the time of day 81, the nude mice were killed and tumor weight was analyzed. Xenograft tumor derived from shRab7a MDA-MB-231 cells exhibited decreased tumor weight (Figure 6C). Collectively, Rab7a silencing inhibits the tumor development *in vivo*.

### Dysregulated gene and signaling pathways in Rab7a knockdown MDA-MB-231 cells

**Figure 7:**
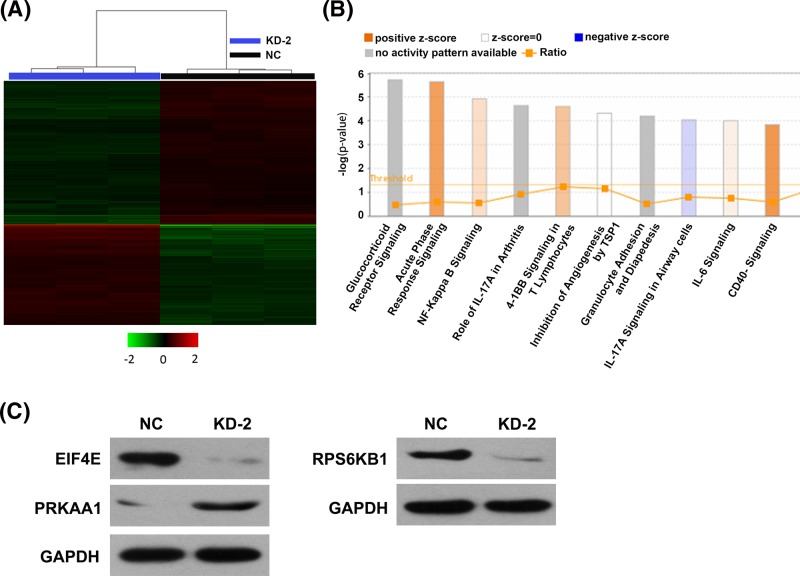
Rab7a silencing causes dysregulation of numerous genes in MDA-MB-231 cells (**A**) Heat map of 634 genes (262 genes up-regulated and 372 genes down-regulated) in MDA-MB-231 cells infected with lentivirus expressing shNC and shRab7a (criteria *P*<0.05, fold-change > 1.5). (**B**) Functional pathway enrichment of dysregulated genes was analyzed by IPA software. (**C**) The expression of eIF4E, PRKAA1, and RPS6KB1 in shNC and shRab7a MDA-MB-231 cells was determined by Western blot assay. GAPDH serves as the internal control.

To explore the correlated molecular mechanisms, dysregulated genes in MDA-MB-231 cells infected with lentivirus expressing shNC or shRab7a was determined using microarray assay. Totally, the expression of 634 genes changed after MDA-MB-231 knockdown (fold-change > 1.5, *P*<0.05), including 262 genes up-regulated and 372 genes down-regulated (Figure 7A). Pathway enrichment analysis revealed that dysregulated genes were enriched in various pathways, including glucocorticoid receptor signaling, dendritic cell maturation, and angiopoietin signaling (Figure 7B). Next, we performed Western blot to validate these results, we found that protein kinase AMP-activated catalytic subunit α 1 (PRKAA1) was up-regulated, and eukaryotic translation initiation factor 4E (eIF4E) and RPS6KB1 were down-regulated after Rab7a knockdown, consistent with the microarray results (Figure 6C). These results indicated that Rab7a might elicit its oncogenic function in breast cancer by dysregulating these genes or pathways.

## Discussion

In the present study, we identified Rab7a as a novel biomarker for breast cancer. Knockdown of Rab7a induced the apoptosis, cell cycle arrest, and promoted the cell proliferation, growth, invasion, and xenografted tumor development of MDA-MB-231 cells. By contrast, Rab7a overexpression reduced the apoptosis and increased the proliferation and colony formation of MCF-7 cells. Mechanically, Rab7a knockdown increased the apoptosis and retarded the cell cycle at S phase.

Breast cancer remains the commonest malignancy in women. Since estrogen and its receptor are closely involved in the development of breast cancer, hormonal therapies against this disease are emerging, such as tamoxifen [4]. This drug following surgery exhibits moderate effectiveness on estrogen receptor positive breast cancer. However, the increased risk of thromboembolism and endometrial cancer caused by estrogen limits its application in breast cancer patients [5]. Therefore, potential diagnosed biomarkers and therapeutic targets are in need for this deadly disease. The primary function of Rab7a is involved in endosomes and lysosomes transport. A previous study reported that Rab7b played an important role in monocytic differentiation of human acute promyelocytic leukemia cells [16]. In this study, we first found that Rab7a was up-regulated in breast cancer tissues and cells. This suggested that Rab7b might participate in breast cancer development. We then constructed four Rab7a knockdown MDA-MB-231 cell lines and found that Rab7a was the most efficiently silenced in KD2 cell lines. Thus, we used KD2 cell lines to investigate the role of Rab7a in breast cancer development. *In vitro*, Rab7a silencing led to suppressed cell proliferation and decreased colony formation of MDA-MB-231 cells. By contrast, the apoptosis was suppressed, and the proliferation and the growth of MCF-7 cells were enhanced by Rab7a overexpression. *In vivo*, Rab7a silencing suppressed the xenograft tumor progression of MDA-MB-231 cells. These findings suggested an essential role of Rab7a in breast cancer development.

It has been reported that Rab7a plays a role in cell migration by regulating Rac1 and vimentin [13]. Vimentin phosphorylation or assembly is also regulated by Rab7a [14]. Since vimentin is biomarker of epithelial–mesenchymal transitions, which play a crucial role in cancer metastasis [17], we attempted to investigate the role of Rab7a in breast cancer cell migration. We showed that Rab7a knockdown blunted the invasion capacity of breast cancer cells. These results suggest that Rab7a is essential for the metastasis of breast cancer. Still, further studies should be performed to address mechanisms of this phenotype.

It is known that cancer cell exhibits a characteristic of accelerated cell cycle. Interphase, containing G_1_, S, and G_2_ phases, is critical for cell growth as the nutrients are prepared during this phase for cell growth [18]. We found that Rab7a silencing largely increased the S-phase but increased the G_2_-phase distribution of cell cycle. This indicated that Rab7a knockdown retarded the cell division at the S-phase. Numerous studies have demonstrated that suppressed apoptosis partly contributes to the uncontrolled cell proliferation of cancer cells. Likewise, we detected increased apoptosis in shRab7a MDA-MB-231 cells. Consistently, decreased apoptosis was observed in Rab7a overexpressing MCF-7 cells. These results might explain why Rab7a silencing or overexpression suppressed or accelerated the proliferation and growth of breast cancer cells, respectively.

Eukaryotic initiation factors (eIFs) have been shown to play an important role in cancer development. mTOR/eIF4E axis is found to contribute to breast cancer maintenance and progression [19]. In the present study, eIF4E was down-regulated by Rab7a knockdown, suggesting that Rab7a might promote breast cancer through regulating eIF4E. In addition, Rab7a silencing also caused reduced expression of RPS6KB1, which has been reported to be altered in breast cancer tissues [20]. We also showed that PRKAA1 was enhanced by Rab7a depletion. However, little is known about the role of PRKAA1 in cancer development. Thus, further studies should be performed to explore the role of PRKAA1 and RPS6KB1 in breast cancer development.

In summary, our study provided evidences for the first time that Rab7a functioned as an oncogene in breast cancer. Based on lentivirus knockdown strategy, we found that Rab7a silencing led to decreased cell viability, growth, cell invasion, and xenografted tumor growth of MDA-MB-231 cells. Apoptosis and cell cycle were induced by Rab7a knockdown. In addition, Rab7a overexpression caused decreased apoptosis and enhanced proliferation and growth of MCF-7 cells. Overall, Rab7a was critical for breast cancer cell survival and metastasis. Targetting Rab7a might be a potential therapeutic strategy for breast cancer.

## Abbreviations

eIF4E: eukaryotic translation initiation factor 4E
mTOR: mammalian target of rapamycin
AMPK: AMP-activated protein kinase
GAPDH: glyceraldehyde-3-phosphate dehydrogenase
PI: propidium iodide
PRKAA1: protein kinase AMP-activated catalytic subunit α 1
Rab7a: Ras-related protein Rab-7a
RPS6KB1: ribosomal protein S6 kinase B1
