# Expression of Concern: Knockdown of Rab7a suppresses the proliferation, migration and xenograft tumor growth of breast cancer cells

**DOI:** 10.1042/BSR20180480_EOC

**Published:** 2026-08-07

**Authors:** Jiming Xie, Yan Yan, Fang Liu, Hongbin Kang, Fengying Xu, Weili Xiao, Haiyan Wang, Yuzhen Wang

**Affiliations:** 1 Clinical Laboratory, Inner Mongolia People's Hospital, Hohhot 010010, P.R. China; 2 College of Life Science, Inner Mongolia Agricultural University, Hohhot 010018, P.R. China; 3 Department of General Surgery, The Affiliated Hospital of Inner Mongolia Medical University, Hohhot 010010, P.R. China; 4 Inner Mongolia Medical University, Hohhot 010110, P.R. China

**Keywords:** apoptosis, cell cycle arrest, migration, proliferation, Rab7a

*Bioscience Reports* has been made aware of potential issues surrounding the scientific validity of this paper and is hence issuing a permanent Expression of Concern to notify readers. Concerns have been raised regarding the reporting and transparency of ethical approval and human tissue use in this article. The study involved both cancerous and healthy breast tissues; however, the ethics statement does not specify the name of the ethics committee, the approval number, date of approval, or details such as the scope of approval with regards to tissue use, including of healthy breast tissue.

The authors have been contacted regarding these concerns but have not responded to the Journal's queries or concerns raised. As such, the article will remain published but with an associated Expression of Concern.

